# Evaluation of a multiplex real-time PCR targeting the β-tubulin gene for the detection and differentiation of *Sporothrix schenckii* and *Sporothrix brasiliensis*

**DOI:** 10.1128/spectrum.01162-24

**Published:** 2024-10-22

**Authors:** Luisa F. López, Lalitha Gade, Anastasia P. Litvintseva, D. Joseph Sexton

**Affiliations:** 1 Mycotic Diseases Branch, Centers for Disease Control and Prevention, Atlanta, Georgia, USA; University of Wisconsin-Madison, Madison, Wisconsin, USA

**Keywords:** *Sporothrix schenckii*, *Sporothrix brasiliensis*, DNA detection, real-time PCR, FFPE tissues, fresh tissues, isolates

## Abstract

*Sporothrix* sp. is a thermally dimorphic genus of fungi known to cause subacute or chronic subcutaneous lesions in humans and animals and is the cause of increasing public health concern due to spread of feline-associated cases. Here, we adapted and evaluated a recently described real-time PCR assay targeting the β-tubulin gene to rapidly detect and differentiate two related species, *S. schenckii* and *S. brasiliensis*. The assay was tested with 55 *S*. *brasiliensis,* 19 *S*. *schenckii*, and 85 isolates from other clinically relevant fungi, and showed 100% concordance with reference identification methods. The assay showed high analytical sensitivity with a limit of detection of 1 pg of DNA per microliter of sample for both targets. The assay was further evaluated with 11 fresh and 17 formalin-fixed, paraffin-embedded (FFPE) tissues. This multiplex real-time PCR assay successfully detected the DNA from both *S. brasiliensis* and *S. schenckii* isolates as well as *S. schenckii* from fresh and FFPE tissues. Our results demonstrate this assay performs well and could be a helpful molecular tool to support rapid species identification in cultures and primary specimens.

**IMPORTANCE:**

Having available molecular tools to identify and differentiate closely related species will allow clinical, veterinarians, and public health labs to provide diagnostic results with accuracy and short turnaround time for the routine and outbreak response activities.

## INTRODUCTION

*Sporothrix* is a genus of environmental and thermally dimorphic fungi that includes over 50 species, with *S. schenckii* being most known as the causative agent of sporotrichosis, also known as Rose-Gardener’s disease (1). Sporotrichosis can have diverse clinical presentations, including subcutaneous and lymphocutaneous infections, as well as infections of other body regions and organs, such as mucous membranes, lungs, bones, joints, and more (1). Transmission is generally understood to occur via traumatic skin inoculation with contaminated materials or from infected animals (1–3).

*S. brasiliensis* is an emerging and often antifungal-resistant species of *Sporothrix* that is causing increasing public health concern due to high rates of feline-associated zoonotic transmission in feral cat populations, leading to cases in domestic cats, pet owners, and veterinarians (4). *S. brasiliensis* was first described in Brazil but is now spreading into surrounding countries, such as Chile, Uruguay, and Argentina (5–8). To date, the transmission of *S. brasiliensis* in the United States has not been reported. However, the current prevalence of S. *brasiliensis* is not well known because the currently available diagnostic methods cannot readily distinguish closely related species (6, 9).

Currently, recovering an isolate followed by targeted sequencing of the β-tubulin or calmodulin (CAL) gene is the gold standard diagnostic to confirm *Sporothrix brasiliensis* in clinical samples, which is labor intensive, can take weeks, and requires access to sequencing facilities which may not be readily available (1, 2, 10). Other diagnostic methods, such as direct microscopic examinations, serology, and histopathology, can aid the diagnosis of the genus but have shown low sensitivity and cannot reliably distinguish similar species (11–13). At the time of writing, there are no commercially available molecular methods for quick and accurate identification of *Sporothrix* spp. to the species level. However, previous reports in the literature have described molecular-based tools, including conventional PCR, nested PCR, and “rolling circle amplification” reactions, targeting 18S rRNA, β-tubulin, CAL, and chitin synthase 1 genes (14–19). In addition, two real-time PCR assays have been published that were able to differentiate *S. schenckii* from *S. brasiliensis* targeting the β-tubulin (20) and CAL genes (21).

The aim of the present study was to expand upon and evaluate the previously developed real-time PCR assay that targets the β-tubulin gene to detect and differentiate *S. schenckii* and *S. brasiliensis*. We expanded on this assay by incorporating an internal control to detect inhibitors. In addition to testing this assay with isolates, this evaluation also included fresh and formalin-fixed paraffin-embedded (FFPE) primary clinical samples.

## MATERIALS AND METHODS

### Specimens, analytical sensitivity, and analytical specificity

#### Study specimens

A total of 94 specimens including 74 isolates: 55 *S*. *brasiliensis* (12 from cats and 43 from humans) and 19 *S*. *schenckii* (14 from cats and 5 from humans)*,* 11 fresh tissues, and 9 suspected sporotrichosis FFPE tissues from cats were included for the validation. All these specimens were received in the Mycotic Diseases Branch laboratory (MDB) at the U.S. Centers for Disease Control and Prevention (CDC) for routine fungal identification or as part of ongoing fungal disease surveillance.

The panel assessing specificity included 85 isolates from the MDB, CDC, Atlanta, USA collection. This panel included a broad range of clinically relevant fungi such as *Trichophyton* spp. (*n* = 3), *Aspergillus* spp. (*n* = 11), *Fusarium* spp. (*n* = 5), thermally dimorphic molds (*n* = 18), yeasts (*n* = 16), mucormycetes (*n* = 9), and other molds (*n* = 23) (Table S1) and eight FFPE tissue specimens that were previously identified with other pathogens such as *Histoplasma capsulatum, Cryptococcus gattii, Exophiala dermatitidis, Rhizopus oryzae, Fusarium oxysporum, Candida albicans, Aspergillus fumigatus,* and *Mucor circinelloides*.

### DNA extraction and species confirmation

#### Isolates

Isolates were subcultured on Sabouraud dextrose agar plates and incubated at 37°C and 25°C for yeasts and molds, respectively. DNeasy Blood and Tissue Kit (Qiagen, Valencia, CA, USA) was used to perform the DNA extraction from molds and the *Quick*-DNA Fungal/Bacterial Miniprep Kit to extract the genomic DNA from yeasts (Zymo Research, CA, USA), as described by the manufacturer’s instructions. Species confirmation was done by performing conventional PCR (22), using the primers NL1/NL4 for the D1/D2 region of the 28S rRNA gene and ITS5/ITS4 for the ITS1/ITS2 regions as previously described (23, 24), respectively. Sequencing was done following the methodology published by Gade et al. (22), and the data were analyzed using Geneious Prime v.2022.1.1.

### FFPE specimens

DNA was extracted from FFPE specimens using a QIAamp DNA FFPE Advanced Kit (Qiagen, Valencia, CA, USA) by following the manufacturer’s instructions. Species identification was done by conventional PCR as described by Gade et al. (22), using the primers ITS3/ITS4 described by White et al. (23), including amplification conditions, purification of products, sequencing, and data interpretation. Geneious Prime v.2022.1.1 software was used for analysis.

### Fresh tissues

Dneasy Powerlyzer Microbial Kit (Qiagen, Valencia, CA, USA) was used for DNA extraction of freshly ground tissues. The protocol was carried out as described by the manufacturer with some modifications, including 200 µL initial sample volume and 5 min of incubation with the elution buffer. Species confirmation was done by culture and conventional PCR following the protocol described for FFPE specimens.

### Multiplex real-time PCR primers and probes

*Sporothrix* spp. primers and species-specific *S. schenckii* and *S. brasiliensis* probes were used as previously described by Della Terra et al. (20) with some modifications (Table 1). Lambda primers and probe for internal control amplification were used according to Wang et al. (25) protocol with some modifications (Table 1).

**TABLE 1:** Primers and probes used in this study

Primers and probes	Sequence 5′−3′
Sporo-F	CGTCTGAGCGTCTACTTCAACG
Sporo-R	GGACGGCATCCATGGTACC
Probe Sbra	6FAM-CGATCGGCTTTGCTTTGGCCCTAGT-IBQ
Probe Ssch	NED-TCCCACCGTTTGGCAC-MGB-NFQ
Lambda forward	AGCACTGTAAGGTCTATCG
Lambda reverse	CCTGTTGGTTGGGGTAAG
Lambda probe	Cy5-ACCGCCCTATTCTCTCGCTGA-IAbRQSp

### Multiplex real-time PCR assay

The genomic DNA amplification from the specimens was performed by following the protocol described by Della Terra et al. (20) with some modifications for the 20-µL final reaction: 10 µL of TaqMan Genotyping Master Mix (2×) (Thermo Fisher Scientific, USA), Sporo primers each at 0.9 µM final concentration, *S. schenckii* and *S. brasiliensis* probes each at 0.25 µM final concentration, lambda primers each at 0.2 µM final concentration and lambda probe at 0.05 µM final concentration, 2 µL of DNA sample, 1 µL of lambda DNA at 0.3 pg/µL (Thermo Fisher Scientific, USA), and 3.8 µL of sterilized nuclease-free water to complete the final volume.

The cycling conditions on the ABI 7500 FAST system (Applied Biosystems, Thermo Fisher Scientific Inc., Waltham, MA) were as previously described by Della Terra et al. (20) with a cycle modification: first step at 5°C for 2 min, 95°C for 10 min followed by 45 cycles of 95°C for 15 s, and 60°C for 1 min.

#### Controls, replicates, and normalization

Positive genomic DNA from *S. schenckii* B22064 and *S. brasiliensis* B22157 (Table S1), and negative control using nuclease-free water were included in every run. In addition, for clinical specimen processing, both extraction positive control and negative control were tested as well. Lambda DNA was included in every sample as inhibition control. In case of no amplification of the positive controls *S. schenckii* or *S. brasiliensis* and lambda DNA in each genomic DNA, the samples were re-tested.

All samples and controls were run in triplicate, and the median value was used as the result. All DNAs from isolates were normalized to 1 ng/µL.

### Limit of detection

Tenfold serial dilutions from 1 ng/µL to 1 fg/μL of *S. schenckii* and *S. brasiliensis* DNA were tested to establish the limit of detection (LOD). Each dilution was run with 20 replicates. The limit of detection was defined as the lowest concentration the target was detected in 19 of 20 samples.

### Reproducibility

DNAs from four fresh tissues were used to assess the reproducibility of the test. Different aliquots of 100 µL of these samples were kept at −20°C. Then, the DNA was extracted on three different days using one aliquot each time, and the real-time PCRs were run with three technical replicates under the same conditions and following the protocols previously described.

## RESULTS

### Limit of detection

**Fig 1:**
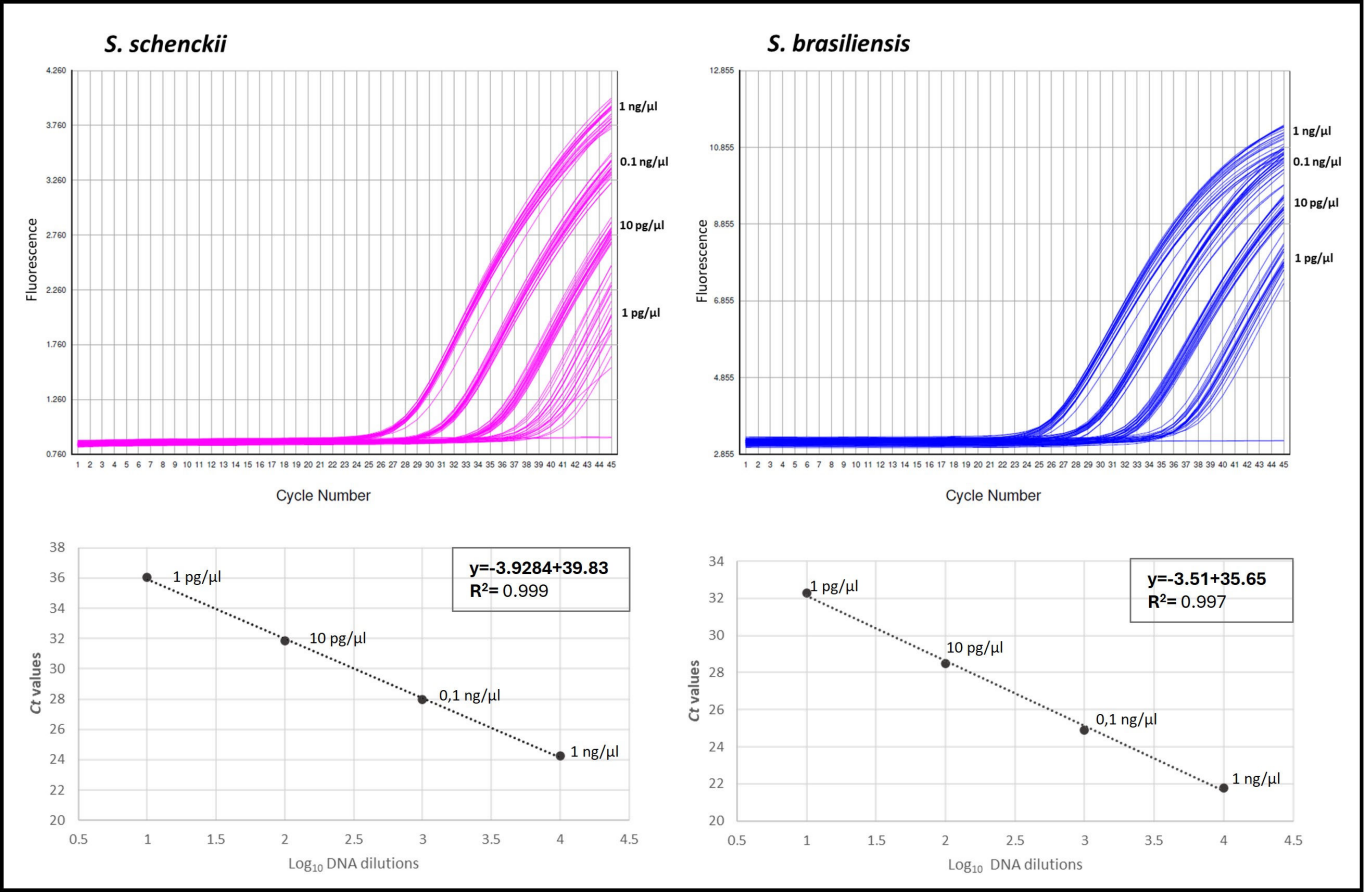
Limit of detection and genomic DNA standard curve of *S. schenckii* and *S. brasiliensis.* Representation of the *S. schenckii and S. brasiliensis* amplifications with genomic DNA concentrations from 1 ng/µL to 1 pg/µL (20 replicates each). Serial 10-fold dilutions of gDNA of *S. schenckii* and *S. brasiliensis* showing the LOD-fit curves with slopes −3.9284 and −3.51, respectively, and the correlation coefficient (*R*^2^). Dots represent the means of the Ct values from the 20 replicates for each dilution.

The LOD of the assay was 1 pg of DNA per microliter of sample. Results of the 20 replicates showed that at this concentration, all the DNAs had a positive signal for both targets (*S. brasiliensis* and *S. schenckii*), and the positivity gradually decreased through the following dilutions (Fig. 1). In addition, this experiment showed a coefficient of variation of <3.3 for each dilution, demonstrating strong reproducibility of the test (Table 2).

**TABLE 2:** LOD multiplex real-time PCR assay results for *S. schenckii* and *S. brasiliensis*

DNA concentration	*S. schenckii*			*S. brasiliensis*		
	Positivity	Average Ct values	Coefficient of variation	Positivity	Average Ct values	Coefficient of variation
1 ng/µL	20/20	24.2	1.51	20/20	21.8	1.64
100 pg/µL	20/20	28	1.33	20/20	24.9	1.66
10 pg/µL	20/20	31.9	1.10	20/20	28.5	1.14
1 pg/µL	20/20	36	3.10	20/20	32.3	2.49
700 fg/µL	12/20	37.6	2.83	18/20	35.4	3.25
400 fg/µL	11/20	37.3	2.04	15/20	35.1	2.60
100 fg/µL	5/20	38.6	2.18	10/20	35.4	1.93
10 fg/µL	1/20	38.6	0.00	1/20	35.4	0.00
1 fg/µL	0/20	NA	NA	0/20	NA^*a*^	NA

^*a*^ NA, not applicable.

### Reproducibility

The results show consistent Ct values over 3 days of the test with the four fresh samples used. The coefficient of variation was <5.0 (Table 3).

**TABLE 3:** Ct values and CV of the reproducibility test using fresh tissues on three different days

Fresh tissue	Ct values			CV^*a*^ (%)
	Day 1	Day 2	Day 3	
1	27.0	29.1	29.4	3.8
2	22.5	24.1	24.6	3.8
3	20.6	20.9	23.0	4.9
4	28.7	30.7	31.5	3.8

^*a*^ CV, coefficient of variation.

### Performance with isolates

All 55 *S*. *brasiliensis* and 19 *S*. *schenckii* DNAs from isolates were correctly amplified and differentiated by the real-time PCR assay with both probes (Table S1) and could be detected at 1 ng/μL. The molecular test was also able to amplify *S. schenckii* DNA from 10 fresh tissues, which were previously confirmed by culture as *S. schenckii*, with Ct values ranging from 19 to 38. The remaining tissue was negative as well as its culture.

### Performance with primary specimens (FFPE and fresh tissues)

Of nine FFPE samples*, S. schenckii* DNA was amplified in six samples with Ct values ranging from 29 to 38 using the multiplex real-time PCR with *Sporothrix* spp. primers and *S. schenckii* and *S. brasiliensis* probes. Three FFPE samples were negative, and the FFPE samples with other fungal identification were also negative for this test. The conventional PCR using the genomic DNA from these nine FFPE samples and primers ITS3/ITS4 (ITS2 region) was able to detect *S. schenckii* DNA in two samples.

Ten fresh tissues from cats were all positive by culture and real-time PCR using *Sporothrix* spp. primers and *S. schenckii* and *S. brasiliensis* probes. Eight of these 10 samples were also positive by conventional PCR using primers ITS3/ITS4 (ITS2 region).

The sequences were deposited in the NCBI database under accession numbers PP933726, PP933728, PP933729, PP933730, PP933731, PP933732, PP933733, and PP933734.

### Analytical specificity

The real-time PCR showed high specificity (100%) and no cross-reactivity as 85 DNAs from closely and distantly related pathogens were tested (Table S2), and no amplification signals were detected.

### Performance of lambda inhibition control

The lambda internal control was amplified with all the DNAs from *S. brasiliensis* and *S. schenckii* isolates as well as fresh tissues. For the specificity panel, the average Ct value was 28, ranging from 27 to 33; the average Ct value for fresh tissue was 32 and for FFPE tissues was 42 (ranging from 41 to 43).

## DISCUSSION

Zoonotic sporotrichosis outbreaks caused by *S. brasiliensis* have been reported widely in Brazil and in neighboring countries. Tracking the continued spread of *S. brasiliensis* is important to inform public health actions but is challenging because there are limited diagnostic options that can rapidly provide a species-level identification (9). In this study, we evaluated a previously described real-time PCR that targets the β-tubulin gene to differentiate *S. schenckii* from *S. brasiliensis*. We further built upon the assay by incorporating an additional internal control designed to detect PCR inhibitors. Overall, we found that this test performed well and could be a valuable method to rapidly differentiate *S. schenckii* and *S. brasiliensis* in clinical and veterinarian specimens as well as a promising tool for public health surveillance.

The performance characteristics of our assay were comparable or exceeded the performances of the other published tests (14, 19–21). The real-time PCR accurately differentiated all *S. brasiliensis* and *S. schenckii* isolates included in this study. The assay also performed well with the primary specimens tested. This finding is encouraging because FFPE samples are a commonly accessible specimen type but are known to be challenging to work with because DNA concentrations are often low and can degrade below the quality required to support Sanger sequencing (22, 26–28). Because real-time PCR does not require as high of DNA quality as Sanger sequencing, this assay could be an attractive option for FFPE samples.

One limitation of this study is the small number of clinical samples and *Sporothrix* species isolates other than *S. schenckii* and *S. brasiliensis*. As a relatively rare disease in the United States, the low number of cases and the absence of a surveillance program (29) made it challenging to obtain desired quantities. For this reason, additional work with more clinical samples and other *Sporothrix* species isolates would be valuable and help lend additional confidence to this assay. However, as our results show, this multiplex real-time PCR is a promising molecular tool to differentiate *S. schenckii* and *S. brasiliensis* as a routine diagnosis test and as a tool for outbreak response.

### Conclusion

We successfully evaluated and adapted a multiplex real-time PCR to detect DNA from *S. brasiliensis* and *S. schenckii* in cultures, fresh, and FFPE tissue specimens. Our study shows that this multiplex molecular tool can provide accuracy, rapid detection, and differentiation between *S. brasiliensis* and *S. schenckii* in a single run in human and animal specimens.

## SUPPLEMENTAL MATERIAL

Tables S1 and S2 spectrum.01162-24-s0001.pdf Isolates used for the specificity test.

ASM does not own the copyrights to Supplemental Material that may be linked to, or accessed through, an article. The authors have granted ASM a non-exclusive, world-wide license to publish the Supplemental Material files. Please contact the corresponding author directly for reuse.

## References

[1] Orofino-Costa R, Macedo PM de, Rodrigues AM, Bernardes-Engemann AR. 2017. Sporotrichosis: an update on epidemiology, etiopathogenesis, laboratory and clinical therapeutics. An Bras Dermatol 92:606–620. doi:10.1590/abd1806-4841.2017279 29166494 PMC5674690

[2] de Carvalho JA, Monteiro RC, Hagen F, Camargo ZP de, Rodrigues AM. 2022. Trends in molecular diagnostics and genotyping tools applied for emerging Sporothrix species. J Fungi (Basel) 8:809. doi:10.3390/jof8080809 36012797 PMC9409836

[3] Gremião IDF, Martins da Silva da Rocha E, Montenegro H, Carneiro AJB, Xavier MO, de Farias MR, Monti F, Mansho W, de Macedo Assunção Pereira RH, Pereira SA, Lopes-Bezerra LM. 2021. Guideline for the management of feline sporotrichosis caused by Sporothrix brasiliensis and literature revision. Braz J Microbiol 52:107–124. doi:10.1007/s42770-020-00365-3 32990922 PMC7966609

[4] Ribeiro Dos Santos A, Gade L, Misas E, Litvintseva AP, Nunnally NS, Parnell LA, Rajeev M, de Souza Carvalho Melhem M, Takahashi JPF, Oliboni GM, Bonfieti LX, Araujo LS, Cappellano P, Venturini J, Lockhart SR, Sexton DJ. 2024. Bimodal distribution of azole susceptibility in Sporothrix brasiliensis isolates in Brazil. Antimicrob Agents Chemother 68:e0162023. doi:10.1128/aac.01620-23 38385701 PMC10989022

[5] Rodrigues AM, de Melo Teixeira M, de Hoog GS, Schubach TMP, Pereira SA, Fernandes GF, Bezerra LML, Felipe MS, de Camargo ZP. 2013. Phylogenetic analysis reveals a high prevalence of Sporothrix brasiliensis in feline sporotrichosis outbreaks. PLoS Negl Trop Dis 7:e2281. doi:10.1371/journal.pntd.0002281 23818999 PMC3688539

[6] Sanchotene KO, Madrid IM, Klafke GB, Bergamashi M, Della Terra PP, Rodrigues AM, de Camargo ZP, Xavier MO. 2015. Sporothrix brasiliensis outbreaks and the rapid emergence of feline sporotrichosis. Mycoses 58:652–658. doi:10.1111/myc.12414 26404561

[7] García Duarte JM, Wattiez Acosta VR, Fornerón Viera PML, Aldama Caballero A, Gorostiaga Matiauda GA. 2017. Esporotricosis trasmitida por gato doméstico. Reporte de un caso familiar. Rev Nac 9:67–76. doi:10.18004/rdn2017.0009.02.067-076

[8] Etchecopaz AN, Lanza N, Toscanini MA, Devoto TB, Pola SJ, Daneri GL, Iovannitti CA, Cuestas ML. 2020. Sporotrichosis caused by Sporothrix brasiliensis in Argentina: case report, molecular identification and in vitro susceptibility pattern to antifungal drugs. J Mycol Med 30:100908. doi:10.1016/j.mycmed.2019.100908 31732417

[9] Rodrigues AM, de Hoog GS, de Camargo ZP. 2016. Sporothrix species causing outbreaks in animals and humans driven by animal–animal transmission. PLoS Pathog 12:e1005638. doi:10.1371/journal.ppat.1005638 27415796 PMC4945023

[10] Kauffman CA, Bustamante B, Chapman SW, Pappas PG, Infectious Diseases Society of America. 2007. Clinical practice guidelines for the management of sporotrichosis: 2007 update by the infectious diseases society of America. Clin Infect Dis 45:1255–1265. doi:10.1086/522765 17968818

[11] Oyarce JA, García C, Alave J, Bustamante B. 2016. Epidemiological clinical and laboratory characterization of sporotrichosis in patients of a tertiary care hospital in Lima, Peru, from 1991 to 2014. Rev Chilena Infectol 33:315–321. doi:10.4067/S0716-10182016000300012 27598283

[12] Bernardes-Engemann AR, Costa RCO, Miguens BR, Penha CVL, Neves E, Pereira BAS, Dias CMP, Mattos M, Gutierrez MC, Schubach A, Oliveira Neto MP, Lazéra M, Lopes-Bezerra LM. 2005. Development of an enzyme-linked immunosorbent assay for the serodiagnosis of several clinical forms of sporotrichosis. Med Mycol 43:487–493. doi:10.1080/13693780400019909 16320492

[13] Almeida-Paes R, Pimenta MA, Pizzini CV, Monteiro PCF, Peralta JM, Nosanchuk JD, Zancopé-Oliveira RM. 2007. Use of mycelial-phase Sporothrix schenckii exoantigens in an enzyme-linked immunosorbent assay for diagnosis of sporotrichosis by antibody detection. Clin Vaccine Immunol 14:244–249. doi:10.1128/CVI.00430-06 17215334 PMC1828849

[14] Hu S, Chung W-H, Hung S-I, Ho H-C, Wang Z-W, Chen C-H, Lu S-C, Kuo T-T, Hong H-S. 2003. Detection of Sporothrix schenckii in clinical samples by a nested PCR assay. J Clin Microbiol 41:1414–1418. doi:10.1128/JCM.41.4.1414-1418.2003 12682123 PMC153868

[15] Indoung S, Chanchayanon B, Chaisut M, Buapeth K, Morteh R, Jantrakajorn S. 2022. Feline sporotrichosis caused by Sporothrix schenckii sensu stricto in Southern Thailand: phenotypic characterization, molecular identification, and antifungal susceptibility. Med Mycol Open Access 60:myac075. doi:10.1093/mmy/myac075 36130102

[16] Yu X, Wan Z, Zhang Z, Li F, Li R, Liu X. 2013. Phenotypic and molecular identification of Sporothrix isolates of clinical origin in Northeast China. Mycopathologia 176:67–74. doi:10.1007/s11046-013-9668-6 23771481 PMC3731519

[17] Kano R, Watanabe K, Murakami M, Yanai T, Hasegawa A. 2005. Molecular diagnosis of feline sporotrichosis. Vet Rec 156:484–485. doi:10.1136/vr.156.15.484 15828746

[18] Rodrigues AM, de Hoog GS, de Camargo ZP. 2015. Molecular diagnosis of pathogenic Sporothrix species. PLoS Negl Trop Dis 9:e0004190. doi:10.1371/journal.pntd.0004190 26623643 PMC4666615

[19] Luiz RLF, Menezes RC, Pereira SA, de Oliveira R de V, Oliveira MME. 2021. Nested PCR for the diagnosis of feline sporotrichosis from formalin-fixed and paraffin-embedded samples using different DNA extraction protocols. Front Vet Sci 8:755897. doi:10.3389/fvets.2021.755897 35071377 PMC8766819

[20] Della Terra PP, Gonsales FF, de Carvalho JA, Hagen F, Kano R, Bonifaz A, Camargo ZP de, Rodrigues AM. 2022. Development and evaluation of a multiplex qPCR assay for rapid diagnostics of emerging sporotrichosis. Transbound Emerg Dis 69:e704–e716. doi:10.1111/tbed.14350 34687495

[21] Zhang M, Li F, Li R, Gong J, Zhao F. 2019. Fast diagnosis of sporotrichosis caused by Sporothrix globosa, Sporothrix schenckii, and Sporothrix brasiliensis based on multiplex real-time PCR. PLoS Negl Trop Dis 13:e0007219. doi:10.1371/journal.pntd.0007219 30817761 PMC6394905

[22] Gade L, Hurst S, Balajee SA, Lockhart SR, Litvintseva AP. 2017. Detection of mucormycetes and other pathogenic fungi in formalin fixed paraffin embedded and fresh tissues using the extended region of 28S rDNA. Med Myco 55:myw083. doi:10.1093/mmy/myw083 27630252

[23] White TJ, Bruns T, Lee S, Taylor J. 1990. Amplification and direct sequencing of fungal ribosomal RNA genes for phylogenetics, p 315–322. In Innis MA, Gelfand DH, Sninsky JJ, White TJ (ed), PCR protocols: a guide to methods and applications. Academic Press, Inc, San Diego, CA.

[24] Voigt K, Cigelnik E, O’donnell K. 1999. Phylogeny and PCR identification of clinically important Zygomycetes based on nuclear ribosomal-DNA sequence data. J Clin Microbiol 37:3957–3964. doi:10.1128/JCM.37.12.3957-3964.1999 10565914 PMC85855

[25] Wang H-Y, Lu J-J, Chang C-Y, Chou W-P, Hsieh JC-H, Lin C-R, Wu M-H. 2019. Development of a high sensitivity TaqMan-based PCR assay for the specific detection of Mycobacterium tuberculosis complex in both pulmonary and extrapulmonary specimens. Sci Rep 9:113. doi:10.1038/s41598-018-33804-1 30643154 PMC6331544

[26] Gade L, Grgurich DE, Kerkering TM, Brandt ME, Litvintseva AP. 2015. Utility of real-time PCR for detection of Exserohilum rostratum in body and tissue fluids during the multistate outbreak of fungal meningitis and other infections. J Clin Microbiol 53:618–625. doi:10.1128/JCM.02443-14 25520443 PMC4298529

[27] Bass BP, Engel KB, Greytak SR, Moore HM. 2014. A review of preanalytical factors affecting molecular, protein, and morphological analysis of formalin-fixed, paraffin-embedded (FFPE) tissue: how well do you know your FFPE specimen? Arch Pathol Lab Med 138:1520–1530. doi:10.5858/arpa.2013-0691-RA 25357115

[28] López LF, Tobón ÁM, Cáceres DH, Chiller T, Litvintseva AP, Gade L, González Á, Gómez BL. 2023. Application of real-time PCR assays for the diagnosis of histoplasmosis in human FFPE tissues using three molecular targets. J Fungi (Basel) 9:700. doi:10.3390/jof9070700 37504689 PMC10381543

[29] Rees JR, Pinner RW, Hajjeh RA, Brandt ME, Reingold AL. 1998. The epidemiological features of invasive mycotic infections in the San Francisco Bay area, 1992-1993: results of population-based laboratory active surveillance. Clin Infect Dis 27:1138–1147. doi:10.1093/clinids/27.5.1138 9827260

