# Supplementary material for: Evaluation of a multiplex real-time PCR targeting the β-tubulin gene for the detection and differentiation of Sporothrix schenckii and Sporothrix brasiliensis
Source: Microbiol Spectr. 2024 Oct 22;12(12):e01162-24. doi: 10.1128/spectrum.01162-24 (PMC11619315; doi:10.1128/spectrum.01162-24)
Supplement: Tables S1 and S2 — Isolates used for the specificity test. [file spectrum.01162-24-s0001.pdf]

S1 Table. *Ct v*alues using DNAs from *S. schenckii* and *S. brasiliensis* isolates

		***S. schenckii* probe**		***S. brasiliensis* probe**	
		***Ct* value**	***SD***	***Ct* value**	***SD***
***S. schenckii* isolates**	B10282	22	0.04	no signal (>45)	NA
	B11252	24	1.1	no signal (>45)	NA
	B22064	24	0.1	no signal (>45)	NA
	B22065	24	0.5	no signal (>45)	NA
	B22103	23	0.06	no signal (>45)	NA
	B22147	23	0.04	no signal (>45)	NA
	Vt-01	24	0.2	no signal (>45)	NA
	Vt-02	22	0.08	no signal (>45)	NA
	Vt-03	24	0.2	no signal (>45)	NA
	Vt-04	23	0.03	no signal (>45)	NA
	Vt-05	23	0.06	no signal (>45)	NA
	Vt-06	20	0.1	no signal (>45)	NA
	Vt-07	20	0.1	no signal (>45)	NA
	Vt-08	20	0.09	no signal (>45)	NA
	Vt-09	20	0.2	no signal (>45)	NA
	Vt-10	20	0.1	no signal (>45)	NA
	Vt-11	20	0.1	no signal (>45)	NA
	Vt-12	19	0.1	no signal (>45)	NA
	Vt-13	20	0.2	no signal (>45)	NA
***S. brasiliensis* isolates**	B22061	no signal (>45)	NA	22	0.07
	B22062	no signal (>45)	NA	22	0.1
	B22063	no signal (>45)	NA	23	0.4
	B22066	no signal (>45)	NA	23	0.1
	B22067	no signal (>45)	NA	22	0.1
	B22069	no signal (>45)	NA	22	0.1
	B22070	no signal (>45)	NA	22	0.06
	B22071	no signal (>45)	NA	22	0.1
	B22072	no signal (>45)	NA	22	0.03
	B22073	no signal (>45)	NA	24	0.1
	B22074	no signal (>45)	NA	23	0.03
	B22077	no signal (>45)	NA	24	0.01
	B22079	no signal (>45)	NA	22	0.3
	B22080	no signal (>45)	NA	24	0.05
	B22081	no signal (>45)	NA	23	0.07
	B22083	no signal (>45)	NA	23	0.05
	B22084	no signal (>45)	NA	23	0.09
	B22085	no signal (>45)	NA	23	0.1

B22086	no signal (>45)	NA	22	0.08
B22087	no signal (>45)	NA	23	0.07
B22088	no signal (>45)	NA	23	0.7
B22089	no signal (>45)	NA	23	0.06
B22090	no signal (>45)	NA	22	0.1
B22091	no signal (>45)	NA	22	1.4
B22092	no signal (>45)	NA	24	0.04
B22093	no signal (>45)	NA	23	0.2
B22094	no signal (>45)	NA	23	0.3
B22095	no signal (>45)	NA	22	0.1
B22096	no signal (>45)	NA	23	0.1
B22097	no signal (>45)	NA	23	0.1
B22098	no signal (>45)	NA	23	0.08
B22099	no signal (>45)	NA	22	0.07
B22101	no signal (>45)	NA	22	0.09
B22102	no signal (>45)	NA	23	0.1
B22104	no signal (>45)	NA	23	0.06
B22137	no signal (>45)	NA	23	0.2
B22138	no signal (>45)	NA	22	0.2
B22139	no signal (>45)	NA	23	0.1
B22140	no signal (>45)	NA	22	0.04
B22141	no signal (>45)	NA	22	0.1
B22142	no signal (>45)	NA	21	0.2
B22144	no signal (>45)	NA	22	0.06
B22145	no signal (>45)	NA	22	0.09
B22146	no signal (>45)	NA	22	0.06
B22148	no signal (>45)	NA	23	0.2
B22149	no signal (>45)	NA	22	0.2
B22150	no signal (>45)	NA	23	0.1
B22151	no signal (>45)	NA	22	0.5
B22152	no signal (>45)	NA	23	0.07
B22153	no signal (>45)	NA	22	0.01
B22154	no signal (>45)	NA	22	0.1
B22155	no signal (>45)	NA	22	0.3
B22156	no signal (>45)	NA	21	0.2
B22157	no signal (>45)	NA	22	0.2
B22159	no signal (>45)	NA	22	0.2

Abbreviations: NA - not applicable. SD - standard deviation.

S2 Table. DNAs from Isolates clinically relevant fungi used for the specificity panel

**Isolates**	**Isolates identification**
Tricophyton mentagrophytes	B26039
Tricophyton mentagrophytes	B26060
Tricophyton mentagrophytes	B26071
Fusarium solani	B18854
Fusarium solani	B22247
Fusarium solani	B22248
Fusarium solani	B23941
Fusarium oxysporum	B18944
Aspergillus fumigatus	B22034
Aspergillus fumigatus	B05603
Aspergillus flavus	B06266
Aspergillus flavus	B24030
Aspergillus flavus	B24031
Aspergillus sydowii	B24032
Aspergillus sydowii	B18422
Aspergillus nomiae	B24038
Aspergillus nidulans	B07476
Aspergillus terreus	B07480
Aspergillus niger	B09347
Penicillium species	B18365
Exophiala species	B19331
Exophiala dermatitidis	B19847
Verruconis (Ochroconis) gallopava	B18350
Sporothrix globosa	B22143
Exserohilum rostratum	B18340
Alternaria alternata	B09898
Alternaria species	B21564
Curvularia species	B21329
Fonsecaea pedrosoi	B11521
Cladophialophora bantiana	B21258
Cladosporium species	B18103
Purpureocillium lilacinum	B18349
Purpureocillium lilacinum	B18426
Scedosporium boydii	B23938
Scedosporium apiospermum	B23647
Scedosporium apiospermum	B18417
Lomentospora (Scedosporium) prolificans	B18945
Sarocladium kiliense	B10842
Paecilomyces variotii	B22350

Paecilomyces species	B18124
Trichosporon asahii	B23121
Trichosporon species	B12641
Trichoderma species	B19340
Apophysomyces trapeziformis	B15281
Mucor circinelloides	B19426
Rhizomucor pusillus	B11171
Rhizopus microsporus	B19333
Cunninghamella bertholletiae	B11173
Cunninghamella elegans	B19988
Cunninghamella species	B11291
Lichtheimia ramosa	B17676
Lichtheimia ramosa	B22250
Blastomyces gilchristii	B22024
Blastomyces gilchristii	B22025
Blastomyces dermatitidis	B22128
Blastomyces dermatitidis	B21261
Coccidiodes immitis	B11080
Coccidioides immitis	B22383
Coccidioides immitis	B22377
Coccidioides immitis	B22385
Coccidiodes posadasii	B16712
Coccidioides posadasii	B22379
Coccidioides posadasii	B22376
Coccidioides posadasii	B22387
Coccidioides posadasii	B22388
Histoplasma capsulatum	B16336
Histoplasma capsulatum	B22127
Histoplasma capsulatum	B22131
Histoplasma capsulatum	B22132
Candida auris	B11103
Candida auris	B11220
Candida auris	B11203
Candida haemulonii	B10441
Candida duobushaemulonii	B10440
Candida albicans	B06453
Candida glabrata	B06552
Candida parapsilosis	B06883
Candida lusitaniae	B10448
Candida tropicalis	CAS22-15254
Candida krusei	CAS22-15310
Cryptococcus neoformans	B15271
Cryptococcus gattii species complex	B19067

Malassezia pachydermatis	B19314
Aureobasidium pullulans	B11031
Rhodotorula species	B10148
